# Role of Electrospinning Parameters on Poly(Lactic-co-Glycolic Acid) and Poly(Caprolactone-co-Glycolic acid) Membranes

**DOI:** 10.3390/polym13050695

**Published:** 2021-02-25

**Authors:** María Herrero-Herrero, José Antonio Gómez-Tejedor, Ana Vallés-Lluch

**Affiliations:** 1 Centre for Biomaterials and Tissue Engineering (CBIT), Universitat Politècnica de València, Camino de Vera s/n, 46022 Valencia, Spain; maherhe7@etsii.upv.es (M.H.-H.); jogomez@fis.upv.es (J.A.G.-T.); 2 Biomedical Research Networking Centre in Bioengineering, Biomaterials and Nanomedicine (CIBER-BBN), 46022 Valencia, Spain

**Keywords:** co-polymer, membrane, electrospinning, poly(lactic-co-glycolic acid), poly(caprolactone-co-glycolic acid), PLGA, PCLGA

## Abstract

Poly(lactic-co-glycolic acid) (PLGA) and poly(caprolactone-co-glycolic acid) (PCLGA) solutions were electrospun into membranes with tailored fiber diameter of 1.8 μm. This particular fiber diameter was tuned depending on the used co-polymer by adjusting the electrospinning parameters that mainly influence the fiber diameter. The greatest setting of the fiber diameter was achieved by varying the polymer solution parameters (polymer concentration, solvents and solvents ratio). PLGA was adequately electrospun with 1,1,1,3,3,3-hexafluoro-2-propanol (HFIP), whereas PCLGA required a polar solvent (such as chloroform) with a lower dielectric constant. Moreover, due to the amorphous morphology of PCLGA, pyridine as salt had to be added to the starting solution to increase its conductivity and make it electrospinnable. Indeed, the electrospinning of this co-polymer presents notable difficulties due to its amorphous structure. Interestingly, PCLGA, having a higher glycolic acid molar fraction than commonly electrospun co-polymers (caprolactone:glycolic acid ratio of 45:55 instead of 90:10), could be successfully electrospun, which has not been reported to date. To an accurate setting of fiber diameter, the voltage and the distance from needle to collector were varied. Finally, the study of the surface tension, conductivity and viscosity of the polymer solutions allowed to correlate these particular characteristics of the solutions with the electrospinning variables so that prior knowledge of them enables predicting the required processing conditions.

## 1. Introduction

Electrospinning has been one of the most promising techniques in the tissue engineering and drug delivery fields for the last decades since electrospun nanofibers can mimic the physical structure of the native extracellular matrix (ECM). Also, it allows us to obtain membranes with an extremely high surface-to-volume ratio and tunable porosity in a wide variety of sizes and shapes [1,2,3,4]. Moreover, it is possible to control the properties of the nanofiber membranes (cell affinity, mechanical properties, biodegradability, etc.) and their functionality (i.e., degradation time) by means of two ways: (i) selecting the polymers and using co-polymers or polymer mixtures and (ii) making fibers with a specific fiber diameter [5,6].

Regarding the first way, biodegradable aliphatic polyesters, such as poly(lactic acid) (PLA), poly(glycolic acid) (PGA) and poly(caprolactone) (PCL), are widely used to obtain electrospun membranes. All of them, their co-polymers and their blends have been approved by the FDA for clinical use and are hydrolyzed into non-toxic natural metabolites, being eliminated from the body by the normal physiological processes [7,8]. Interestingly, the shortcomings of homo-polymers can be overcome by their co-polymerization. For example, PLA presents hydrophobicity, poor toughness and lack of reactive side chains, whereas PCL has a quite long degradation time, and PGA degrades quickly in the aqueous medium due to its hydrophilicity [9]. It has been reported a degradation time of 6–12 months for PLA to 2–4 years for PCL, depending on starting crystallinity and molecular weight [10]. Hence, poly(lactic-co-glycolic acid) (PLGA) has a faster biodegradability rate than PLA due to the presence of GA units; in fact, increasing the weight ratio of the GA to LA from 25:75 to 50:50 can accelerate the degradation by two-fold from 100 to 50 days [11]. Poly(caprolactone-co-glycolic acid) (PCLGA) shows more elasticity than PLGA due to the effect of CL [12,13].

Notwithstanding the polymer choice, which will obviously influence the final properties of the electrospun membrane, the parameters of the polymer solutions and the electrospinning process will also determine the fiber diameter and morphology. As we said previously, several properties of the membranes are related to the fiber diameter, so the understanding of the effect of those parameters is key to develop a specific material for a particular application. The most important polymer solution parameters involved in the fiber diameter are the surface tension, the viscosity and the conductivity [13,14]. The effect of the density solution has not been studied much yet because of its relationship with other parameters [15].

Due to the fact that electrospinning uses an electric potential to overcome the surface tension, a reduced surface tension favors the formation of fibers without beads. This parameter is strongly influenced by the nature of the solvent [16,17]. The viscosity is related to the opposition of the jet to be ejected. A too-low viscosity may result in the interruption of polymeric filaments and droplets of polymer, whereas a too-high viscosity makes impossible the extrusion of the polymer. Nevertheless, increasing the viscosity, the diameter of the fiber also increases [17,18,19]. Finally, an increase in the conductivity of the polymer solution entails a decrease in fiber diameter [20].

When the electrospinnability of a solution is low, there are several parameters that can improve it, such as polymer concentration, used solvent or voltage. Nevertheless, if those parameters are not enough, the addition of a salt such as pyridine has been shown to improve it because it increases the conductivity of the polymer solution. The advantage of using pyridine in electrospinning is that it is not necessary to remove it after the process because it evaporates during the solution fly between the needle and the collector [20,21]. Moreover, it is an FDA-approved compound and has already been used as an additive in the pharmaceutical, agricultural or food industries [22].

The main purpose of this study was, thus, to obtain the electrospinning conditions of PLGA and PCLGA co-polymers to achieve fiber membranes thereof with approximately 1.8 µm fiber diameter. We chose this fiber diameter as our goal to allow comparison with the procedure set in previous work for membranes of PLA, PCL and PLA/PCL mixtures. Electrospun membranes with this fiber diameter were found to show the most effective configuration for the release of molecules of interest (here tested with bovine serum albumin (BSA), commonly used as a model release molecule), compared to thinner fibers [23]. Co-polymer solutions were here characterized by the determination of their density, surface tension, viscosity and conductivity to relate with the parameters of the electrospinning process, especially voltage and distance from needle to collector.

The second aim of this article lies in the obtention of PCLGA electrospun membranes having a significant fraction of GA. There are not yet many reports on PCLGA membranes because of the difficulty of electrospinning this highly amorphous co-polymer. Moreover, the standard is based on a monomers molar ratio of 90:10 CL:GA [24], whereas this is the first paper where PCLGA with a molar ratio of 45:55 is successfully electrospun in order to obtain a membrane with intermediate properties between its homo-polymers.

The electrospun membranes developed in this paper could be useful for drug release in different medical fields. Due to their degradability and mechanical properties tailored by their composition, they could be used both inside the body to deliver different drugs and on the skin for wound healing.

## 2. Materials and Methods

### 2.1. Material

Poly(lactic-co-glycolic acid) acid terminated (50:50 M_w_ 38,000–54,000 g/mol), poly(lactic-co-glycolic acid) ester terminated (50:50, M_w_ 38,000–54,000 g/mol) and poly(caprolactone-co-glycolic acid) (caprolactone:glycolic 45:55, viscosity 1.5 dL/g) were purchased from Sigma Aldrich (St. Louis, MI, USA). Chloroform (stabilized with ethanol) was purchased from Scharlab (Barcelona, Spain), and 1,1,1,3,3,3-hexafluoro-2-propanol (≥99%) and pyridine (anhydrous, 99.8%) from Sigma Aldrich.

### 2.2. Polymeric Solutions

Poly(lactic-co-glycolic acid) (PLGA) as a mixture of PLGA acid and PLGA ester terminated at 50% *w/w*, and poly(caprolactone-co-glycolic acid) (PCLGA) were solved in chloroform (chlor) and 1,1,1,3,3,3-hexafluoro-2-propanol (HFIP) at different concentrations. The lower electrospinnability of PCLGA was dealt with by adding pyridine (pyr) to the solution at a concentration of 20% *v/v* with respect to the solvent. Once the solutions were prepared, they were stirred for 48 h before electrospinning.

### 2.3. Electrospinning

A syringe with a needle (24 G, 0.31 mm inner diameter) was filled with the polymeric solution and introduced in a syringe pump (RS-232, model NE 1000. New Era Pump Systems, Inc., Toledo St, Farmingdale, NY. USA) to eject the solution at 2 mL/h. The collector was covered with aluminum foil and placed at different distances (from 13 to 20 cm). A voltage source (OL400W-503, HiTek Power, West Sussex, GB) was used to apply a difference of voltage from 13 kV to 20 kV between the needle and the collector. The process was carried out in a homemade chamber for 5 min, where a dry forced airflow was circulated to maintain the humidity below 20% and to remove solvent vapors at room temperature (between 20 and 22 °C). The electrospinning parameters modified to the optimization of membranes were: polymer concentration (from 10 to 25% *w/v* of PLGA or PCLGA), solvent (chlor, HFIP) and ratio of components in binary solutions (from 5% to 20% *v/v* of pyridine in chlor), voltage (from 13 to 20 kV) and distance from needle to collector (from 13 to 20 cm).

Once electrospun, the membranes were placed overnight in a fume hood to ensure the complete evaporation of solvents. After that, the membranes were separated from the aluminum foil and stored in a fridge (4 °C) until characterization to avoid degradation, which is faster at room temperature. Three 10 cm × 15 cm membranes were obtained per conditions set.

### 2.4. Scanning Electron Microscopy (SEM)

To determine the electrospinning parameters that result in membranes of 1.8 μm without defects, such as beads, the surface exposed to the air of the membranes was first observed in a scanning electron microscope (JEOL model JSM6300, Tokyo, Japan) with a working distance of 12 mm and a voltage of 15 kV. The samples were previously sputtered with gold to make them conductive.

When the SEM images showed an adequate fiber membrane structure without defects, the fiber diameters were assessed using the ImageJ software by measuring the diameter of 50 fibers from two SEM images per material at a magnification of ×1000.

### 2.5. Characterization of the Polymeric Solutions

Once the electrospinning parameters were established, the polymer solutions leading to the optimal membranes were studied in the following terms:

#### 2.5.1. Density

A pycnometer of 10 mL was used to quantify the density of the polymeric solutions. The volume was calibrated with distilled water as reference liquid, from which density could be estimated by the following formula [25]:

(1) $$\begin{array}{l} \rho_{w}=(999.85308+6.32693\cdot 10^{-2}\left(\frac{t}{{}^{\circ}C}\right)-8.523829\cdot 10^{-3}{\left(\frac{t}{{}^{\circ}C}\right)}^{2}+6.943248\cdot 10^{-5}{\left(\frac{t}{{}^{\circ}C}\right)}^{3}-3.821216\\ \;\cdot 10^{-7}{\left(\frac{t}{{}^{\circ}C}\right)}^{4})\left(\mathrm{kg}/m^{3}\right) \end{array}$$

where t is the temperature in degrees Celsius.

Weighing the mass of the empty pycnometer ($m_{p}$), the pycnometer filled with distilled water ($m_{p+w}$) and the pycnometer filled with the polymeric solution ($m_{p+s}$), the density of the polymeric solution ($\rho_{s}$) was obtained as:

(2) $$\rho_{s}=\frac{m_{p+s}-m_{p}}{m_{p+w}-m_{p}}\cdot \rho_{w}$$

Three measurements per type of solution were taken.

#### 2.5.2. Surface tension

The polymeric solutions have a high viscosity, and their solvents are highly volatile, so their surface tension was determined with the modified Tate’s law, as described in previous work. Briefly, five drops were weighed using an airtight device to reduce the effect of solvent evaporation. After that, by means of the density of the solutions and applying Tate’s law, the surface tension of the polymeric solution was obtained [23]. Measurements were carried out in triplicate.

#### 2.5.3. Viscosity

To measure the dynamic viscosity of the polymer solutions, an oscillatory assay was performed in a parallel plate’s rheometer (Discovery HR-2 hybrid rheometer, TA Instruments) at an angular frequency of 100 rad/s, resulting in a faster assay that prevents the evaporation of the solvent. 500 µL of polymer solution was measured each time, with 3 replicates per composition.

The complex viscosity ($\eta^{*}$) was obtained from the data of the complex modulus ($G^{*}$) and the angular frequency (ω) as:

(3) $$\eta^{*}=\frac{G^{*}}{\omega }$$

#### 2.5.4. Conductivity

The conductivity of the polymeric solutions was measured per triplicate with a Crison EC-Meter Basic 30+ conductivity meter (HACH LANGE Spain, Barcelona, Spain). The instrument was previously calibrated with three standard solutions, of which conductivity was known.

## 3. Results and Discussion

### 3.1. Determination of the Electrospinning Parameters for PLGA and PCLGA

According to previous work, the main effective parameters to modify fiber diameter are the polymer concentration and the solvent, because they are highly related to viscosity [23]. After that, voltage also plays an important role in fiber diameter. Firstly, the mentioned solution parameters were analyzed. Next, the fiber diameter was set by modifying the voltage. When a more accurate setting was necessary, it was achieved with the modification of the distance from needle to collector and the flow rate.

**Figure 1:**
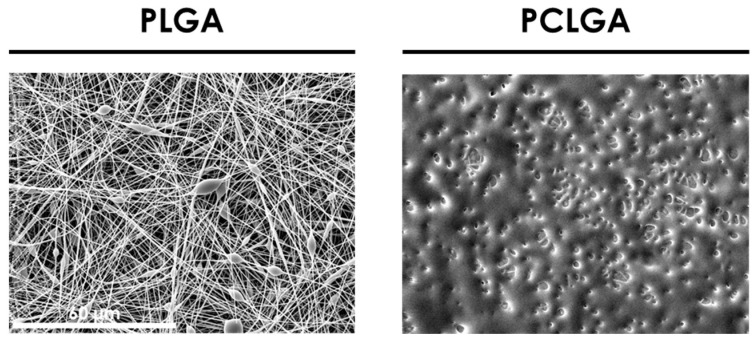
SEM images of Poly(lactic-co-glycolic acid) (PLGA) and poly(caprolactone-co-glycolic acid) (PCLGA) electrospun membranes at 10% *w/v* in 1,1,1,3,3,3-hexafluoro-2-propanol (HFIP), 2 mL/h, 17 kV and 13 cm. Both images share the scale bar: 60 µm.

Considering the results of [26,27], the starting parameters for both co-polymers were established as: a concentration of 10% *w/v* in HFIP with a flow rate of 2 mL/h, a voltage of 17 kV and a distance to collector of 13 cm. The SEM results are shown in Figure 1.

PLGA was adequately electrospun, although it presents beads. Nevertheless, the PCLGA image shows that fibers were not properly formed, probably because the solvent was not evaporated during the electrospinning process.

Due to the different behavior of both co-polymers in the electrospinning, each one was treated specifically: (i) for PLGA, the SEM images of the first approach (Figure 1) showed that an increment in polymer concentration was necessary. For that reason, the concentration was increased up to 25% *w/v*, maintaining HFIP as a solvent. In this way, it was expected that beads disappeared and the fiber diameter increased [17,28]; (ii) for PCLGA, the use of chloroform as solvent was proposed because of its polarity and its lower dielectric constant [29,30]. Moreover, the concentration was also increased up to 20% *w/v*. The electrospinning process parameters were maintained constant to evaluate only the effect of the polymer solution parameters.

**Figure 2:**
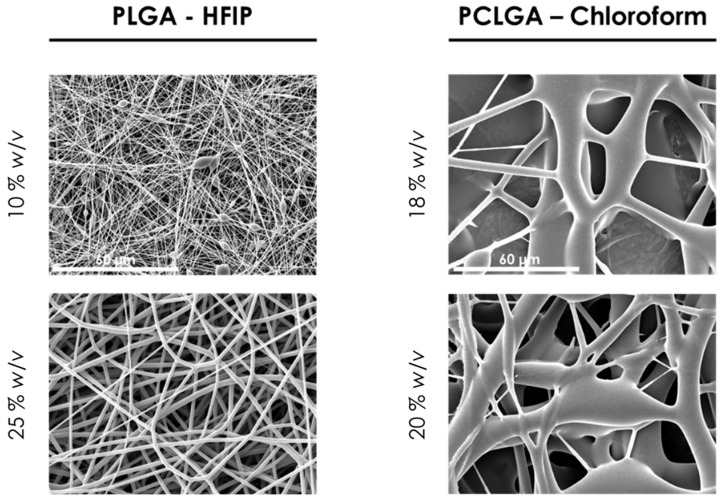
SEM images of PLGA (10 and 25% *w/v* in HFIP) and PCLGA (18 and 20% *w/v* in chloroform) at 2 mL/h, 17 kV and 13 cm. All images share the same scale bar: 60 µm.

As Figure 2 shows, the increment on the polymer concentration for PLGA allowed obtaining homogeneous membranes without defects. Moreover, the diameter analysis determined that the average fiber diameter was 1.62 µm; thus, polymer solution parameters were established at 25% *w/v* in HFIP.

**Figure 3:**
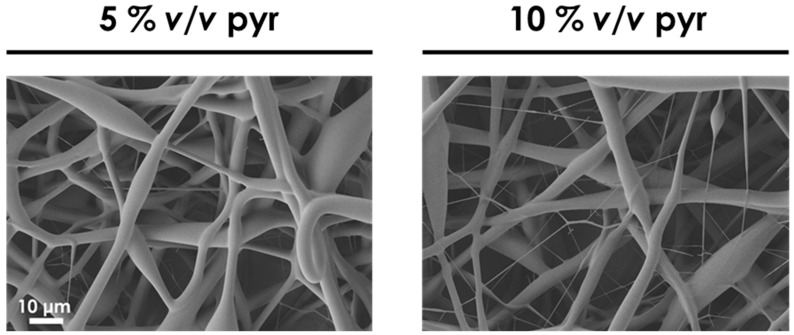
SEM images of 20% *w/v* PCLGA in chloroform with 5% *v/v* and 10% *v/v* of pyridine. 2 mL/h, 15 kV and 20 cm. Both images share the same scale bar: 10 µm.

The use of chloroform with a higher polymer concentration of PCLGA entails the feasibility of electrospinning this co-polymer, although the fibers were not formed adequately. It is well known that a way to obtain uniform and thinner fibers is to increase the conductivity of the polymer solution. Therefore, to achieve finer and uniform fiber diameters, salts can be added to the solution to increase its conductivity [20,31,32]. Thus, pyridine at 5% *v/v* and 10% *v/v* concentration were added to the solution of PCLGA in chloroform at 20% *w/v*, and these polymer solutions were electrospun, maintaining the rest of the electrospinning parameters in 2 mL/h, 15 kV and 20 cm (Figure 3). These amounts of pyridine were selected due to the fact that under 5% *v/v*, the effect on conductivity was not considered remarkable for these membranes, according to [21].

**Figure 4:**
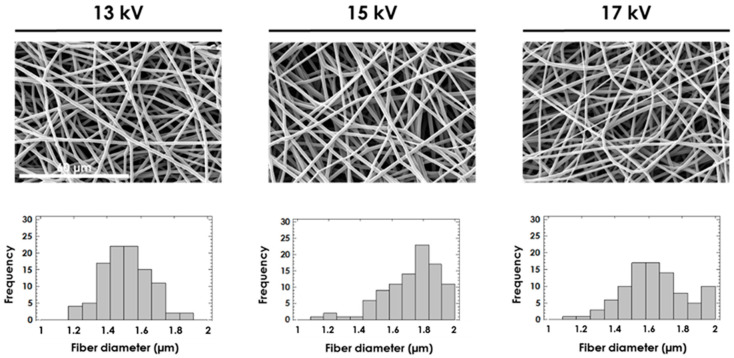
SEM images and histograms of the fiber diameter for 25% *w/v* PLGA in HFIP at 13 kV, 15 kV and 17 kV by maintaining flow rate = 2 mL/h and distance from needle to collector = 13 cm. All images share the same scale bar: 60 µm.

By adding pyridine in the solution at those concentrations, the fibers were much more defined, although the diameter is higher than the required one because of the high concentration of the polymer solution. Therefore, in order to obtain 1.8 µm fibers, the concentration of the polymer solutions was reduced from 20% *w/v* to 17% *w/v*. Moreover, chloroform:pyridine (80:20 *v:v*) was used as a solvent system to improve the effect of pyridine in the process. Once the polymer solution parameters were established, the electrospinning process parameters were modified to an accurate setting of the diameter. Figure 4 shows the effect of voltage in PLGA membranes at 2 mL/h and 13 cm.

The fiber diameter first increased with the applied voltage. Next, it decreased after getting a maximum at an intermediate voltage around 15 kV (1.51 µm average diameter at 13 kV, 1.76 µm at 15 kV and 1.62 µm at 17 kV). This non-monotonic effect of the voltage on the fiber diameter has previously been observed for polymers of a different nature [33,34,35]. It was also observed that the higher the voltage, the higher the variability in fiber diameter (standard deviation: 0.14 µm at 13 kV, 0.20 µm at 15 kV and 0.26 µm at 17 kV). These results were statistically significant and were in agreement with [36]. To sum up, the electrospinning process parameters for PLGA were established as 2 mL/h, 15 kV and 13 cm.

**Figure 5:**
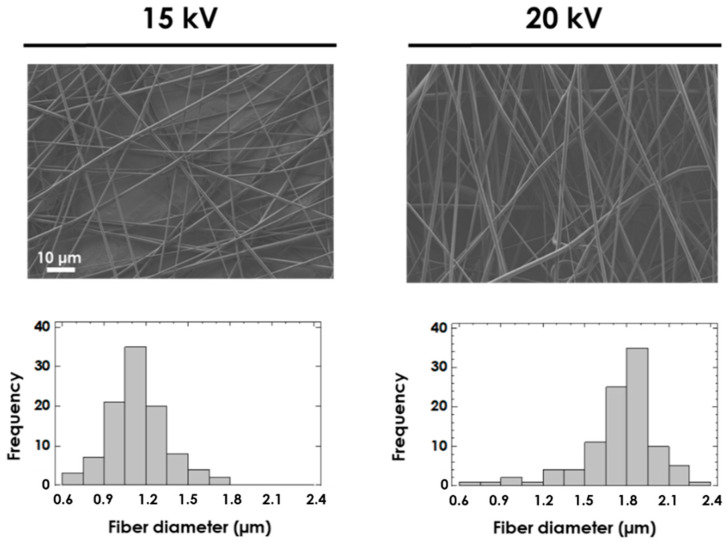
SEM images and histograms of the fiber diameter for 17% *w/v* PCLGA in chloroform:pyridine (80:20 *v:v*) at 15 kV and 20 kV by maintaining flow rate = 2 mL/h and distance from needle to collector = 20 cm. Both images share the same scale bar: 10 µm.

The effect of voltage on PCLGA membranes (Figure 5) was studied by modifying the distance from 13 to 20 cm to reduce the fiber diameter in order to achieve a greater elongation of fibers. As occurred with PLGA, the diameter of electrospun fibers increases at higher voltages: (1.15 ± 0.21) µm at 15 kV and (1.74 ± 0.28) µm at 20 kV. According to these results, the electrospinning conditions of PCLGA to obtain homogeneous fibers of approximately 1.8 µm were: 17% *w/v* PCLGA in chloroform:pyridine (80:20 *v:v*), 2 mL/h, 20 kV and 20 cm.

In Table 1, the electrospinning conditions for both co-polymers are summarized, together with the resulting fiber diameter, much closer to the objective of 1.8 µm (1.76 µm for PLGA and 1.74 µm for PCLGA).

### 3.2. Characterization of the Solution Parameters: Density, Surface Tension, Viscosity and Conductivity

The polymer solutions of Table 1 were next characterized in terms of density, surface tension, viscosity and conductivity. The densities were (1.258 ± 0.010) g/mL for that of PLGA and (1.018 ± 0.006) g/mL for PCLGA (statistically significant). This difference can be attributed, on the one hand, to the higher concentration of the PLGA solution, which increases its density. On the other hand, HFIP has a higher density than chloroform and pyridine, and therefore their mixtures.

In general, density has a moderate influence on fiber diameter, and there are very few studies assessing its effect on fiber morphology. In an electrospinning theoretical model [15], as well as in [37], it was found that the increase in the density implies a slight decrease in the fiber diameter. For that reason, the applied voltage and distance from needle to collector of the PCLGA solution are higher than those of PLGA.

Co-polymer solutions of Table 1 showed the following surface tensions: (158.2 ± 0.5) mN/m for PLGA and (136.8 ± 0.9) mN/m for PCLGA (statistically significant). The higher surface tension of the PLGA solution results in a slightly greater opposition of this solution to be ejected [17]. For this reason, the solvent could evaporate faster, and the distance from needle to collector can be reduced from 20 cm for PCLGA to 13 cm for PLGA.

As for the values of storage (*G*′) and loss (*G*″) moduli, tan (*δ*), dynamic (*η*′) and storage (*η*″) viscosities of the solutions, they are shown in Table 2. The storage modulus (*G*′) represents the energy stored in the elastic structure of the sample, whereas the loss modulus (*G*″) represents the viscous part of the amount of energy dissipated thereof [38]. Therefore, plasticity is related to the loss modulus and elasticity of the storage modulus. Plasticity contributes to nanofiber formation, and elasticity is critical in the stage of jet formation and elongation, preventing the jet from breaking up. A higher *G*′ results consequently in a beadless nanofiber structure [39]. In this case, both co-polymer solutions show a higher storage modulus than the loss one, it being greater for PCLGA.

Nevertheless, the dynamic viscosity (*η*’) of PLGA is higher than PCLGA, although there is no statistical significance. However, it has been reported that the addition of pyridine decreases the dynamic viscosity [22]. Moreover, a higher dynamic viscosity entails a more uniform distribution of the nanofibers, as can be observed in Table 2 for PLGA.

As for their conductivity, the PLGA solution yielded a value of (0.98 ± 0.01) S/cm, whereas that of PCLGA was (0.31 ± 0.01) S/cm (statistically significant). This latter lower conductivity explains the need for a higher voltage for the PCLGA solution to make it electrospinnable. In this way, a higher conductivity exhibits an easier accumulation of charges in the solution [40]. The features of the polymeric solutions of Table 1 are summarized in Table 3.

## 4. Conclusions

PCLGA with a CL:GA molar ratio of 45:55 (far from the commonly processed ratio of 90:10) was properly electrospun, this co-polymer being of interest in the tissue engineering field for its intermediate properties between PCL and PGA. Moreover, membranes of PLGA and PCLGA were successfully obtained with comparable fiber diameters of approximately 1.8 µm. PLGA is adequately electrospun in HFIP, although a high polymer concentration (25% *w/v*) is necessary to avoid the presence of beads; nevertheless, the polymer/solvent system of PCLGA/HFIP is not electrospinnable, probably due to the polarity of the solvent. For that reason, for PCLGA, the use of chloroform as a solvent is more appropriate to obtain electrospun membranes, the addition of salt (such as pyridine) being also required in this case. The higher surface tension of the PLGA solution allowed reducing the distance between the needle and the collector. On the contrary, the low conductivity of PCLGA required the increment of the voltage.

All in all, these findings indicate that the properties of the polymer solutions, modulated in this case by the concentration and the solvent, play a main role in the morphology of their membranes. The advantage of knowing them can be taken to tune other electrospinning parameters such as voltage and distance to collector in order to obtain membranes with a targeted fiber diameter.

## Figures and Tables

**Table 1:** Concentration and solvents of the PLGA and PCLGA solutions and electrospinning parameters to obtain membranes of 1.8 µm thereof.

	PLGA	PCLGA
Fiber diameter (µm)	1.76 ± 0.20	1.74 ± 0.28
Polymer concentration (% *w/v*)	25	17
Solvent	HFIP	chloroform:pyridine (80:20 *v:v*)
Flow rate (mL/h)	2	2
Voltage (kV)	15	20
Distance needle to collector (cm)	13	20

**Table 2:** Storage (*G*′) and loss (*G*″) moduli, tan (*δ*), dynamic (*η*′) and storage (*η*″) viscosities for PLGA and PCLGA solutions described in Table 1, measured at 100 rad/s.

	PLGA	PCLGA
*G*′ (Pa)	901.5 ± 1.6	1180.0 ± 90.4
*G*″ (Pa)	259.5 ± 6.5	232.4 ± 25.8
tan (*δ*)	0.288 ± 0.008	0.197 ± 0.020
*η*′ (Pa·s)	2.595 ± 0.065	2.324 ± 0.258
*η*″ (Pa·s)	9.015 ± 0.016	11.798 ± 0.904

**Table 3:** Physico-chemical properties of the polymeric solutions presented in Table 1.

	PLGA	PCLGA
Density (g/mL)	1.258 ± 0.010	1.018 ± 0.006
Surface tension (mN/m)	158.2 ± 0.5	136.8 ± 0.9
Conductivity (S/cm)	0.98 ± 0.01	0.31 ± 0.01
Dynamic viscosity (Pa·s)	2.595 ± 0.065	2.324 ± 0.256

## Data Availability

The data presented in this study are available on request from the corresponding author.
